# Resistant Starch Production and Glucose Release from Pre‐Prepared Chilled Food: The SPUD Project

**DOI:** 10.1111/nbu.12476

**Published:** 2020-11-22

**Authors:** T. M. Robertson, J. E. Brown, B. A. Fielding, R. Hovorka, M. D. Robertson

**Affiliations:** ^1^ University of Surrey Guildford UK; ^2^ University of Cambridge Cambridge UK

**Keywords:** food processing, glucose metabolism, glycaemic response, stable isotopes, starch

## Abstract

With an increasing prevalence of diabetes worldwide, effective dietary strategies for blood glucose control are crucial. As carbohydrates make up approximately 50% of the diet, it is neither practical nor advisable to avoid them altogether. Most of the carbohydrate in the diet is derived from starch, found in potatoes, pasta, rice and bread. These foods are often processed in some way before consumption, yet little is known about the effects processing, such as chilling and reheating, has on the glycaemic response, particularly when the food is consumed in the context of a mixed meal. This article introduces the *SPUD* project, a BBSRC DRINC‐funded initiative. Taking the potato as the model carbohydrate, this project will investigate, via *in vitro* and *in vivo* studies, the effects of domestic food processing techniques on the glycaemic response. A final study, utilising intrinsically labelled potato and a dual stable isotope methodology, will model glucose flux data to determine the underlying mechanisms of action.

## Introduction

Diabetes is a non‐communicable disease characterised by the body’s inability to control blood glucose effectively. It is usually classified into two types: type 1, an autoimmune disorder; and type 2 (T2D) which is linked to obesity and tends to develop later in life. However, with the rise in childhood obesity, cases in younger people are becoming more common. Prevalence of both types is increasing (Centers for Disease Control & Prevention 2020), with current projections suggesting that 700 million people worldwide will be affected, with associated global health costs of USD 845 billion, by 2045 (International Diabetes Federation 2019). In healthy subjects, the consumption of carbohydrate (CHO)‐containing foods causes a temporary increase in blood glucose, known as the postprandial glycaemic response. In response, the pancreas secretes insulin, causing cells to take glucose from the blood, returning blood glucose concentration to pre‐meal levels, usually within 3 hours. For people with diabetes, however, the postprandial glucose excursion can be elevated and prolonged, unless controlled by medication or diet and lifestyle changes. Prolonged hyperglycaemia is associated with long‐term complications of diabetes, such as heart disease, stroke, peripheral neuropathy, peripheral vascular disease and retinopathy. Glycaemic index (GI) and glycaemic load (GL) are measures of how much a CHO‐containing food raises blood glucose, with high GI and/or high GL diets associated with future development of T2D in both men and women (Salmerón *et al*. 1997a, 1997b). Minimising the postprandial glucose excursion is therefore important, not just for those living with diabetes, but also for the population as a whole.

## Carbohydrate types and glycaemia

Carbohydrates can be classified into three broad categories: sugars, starches and fibre. Sugars can be subdivided into mono‐ and disaccharides; monosaccharides, such as glucose, fructose and galactose, are absorbed directly from the small intestine to the blood. Disaccharides are composed of two monosaccharide subunits, for example sucrose (table sugar) is formed from glucose and fructose. Disaccharides and starches are broken down into monosaccharides by enzymes in the small intestine before being taken up into the cells and transported into the blood. Fibre is not digested in the small intestine, but passes to the colon, where it is fermented by gut bacteria into short‐chain fatty acids (Byrne *et al*. 2015). It does not contribute to the postprandial glucose excursion.

Sugars have the simplest chemical structures and produce the fastest rise in blood glucose. Starches, found in foods such as bread, pasta and potatoes, have a more complex structure containing long polymers of glucose subunits: amylose, which has a linear structure and amylopectin, which is branched. Amylose is digested more slowly than amylopectin. Starchy foods contain a mix of amylopectin and amylose molecules, with the amylose–amylopectin ratio differing between foods. Starch structures take longer to break down and so consequently tend to produce a slower rise in blood glucose. However, this is also affected by the level of processing (Fardet *et al*. 2006), with more processed foods, such as white bread, producing a faster glycaemic response, akin to that exhibited by table sugar. Starches can be classified according to how quickly they are digested: rapidly digestible starch (RDS), slowly digestible starch (SDS) and resistant starch (RS), which is classed as a dietary fibre. There are five types of RS, which are detailed in Table 1.

**Table 1:** Types of resistant starch

Type	Description
RS1	Physically inaccessible: starch is trapped within the food matrix (e.g. beans, lentils)
RS2	Crystalline structure: found in raw foods, such as unripe bananas
RS3	Retrograded starch: forms after cooked gelatinised starch cools (e.g. cooked and cooled potatoes or pasta)
RS4	Chemically modified starch (e.g. by esterification or cross‐linking)
RS5	Amylose–lipid complex: formed during processing with lipid

RS, resistant starch.

The different starch fractions can be measured by incubating a sample of the test CHO with a mix of digestive enzymes, including invertase, pancreatin and amyloglucosidase (Englyst & Hudson 1996). Rapidly available glucose (RAG) is defined as the amount of glucose released after 20 minutes incubation; RDS can be derived from RAG by correcting for free glucose originally in the sample. Both RAG and RDS have a large effect on postprandial glucose and are closely correlated with GI (Englyst *et al*. 1996). SDS is defined as the amount of glucose released between 20 and 120 minutes; it has a much smaller effect on the glycaemic response than RDS. RS can be calculated as the difference between total starch and the amount of glucose released after 120‐minute incubation; it does not contribute to the postprandial glucose excursion (Englyst & Hudson 1996).

Substitution of some of the available CHO in a meal, with RS, results in an attenuation of the postprandial glycaemic response (Behall & Hallfrisch 2002). The evidence for this effect is sufficiently strong that there is an European Food Safety Authority (EFSA)‐approved health claim for a reduction in glycaemic response provided at least 14% of the available CHO in a meal is replaced with RS (Agostoni *et al*. 2011). Chronic intervention studies have also demonstrated beneficial effects of RS on glucose metabolism; supplementation with RS over a 4‐week period resulted in increased glucose clearance and improved insulin sensitivity during a mixed meal tolerance test in comparison with a control group (Robertson *et al*. 2005). Other health effects of RS, such as its effects on gut and colon health, have been comprehensively reviewed elsewhere (Lockyer & Nugent 2017).

## Glycaemic Index (GI) and Glycaemic Load (GL)

The GI is a ranking from 1‐100, relating to how much a CHO‐containing food causes blood glucose to rise. It is a metric associated with the carbohydrate‐rich food, although it is derived from the human physiological response to consumption of that food. It is quantified by feeding a minimum of 10 people a portion of the test food that contains 50 g digestible CHO, and measuring the incremental area under their blood glucose response curve (IAUC) for 2 hours postprandially. The same group of people, on a separate occasion, consume 50 g CHO from a reference food, either sugar or white bread, and the 2 hour IAUC for the reference food is also measured. The test food is consumed twice and the reference food three times per person, and the mean IAUC for each is calculated. The GI is then calculated as IAUC (test food)/IAUC (reference food) x 100 (Brouns *et al*. 2005). Whilst it allows different foods to be directly compared, based on a 50 g CHO portion, it does not take account of the amount of CHO in a standard portion.

A given food may have a high GI but contain a relatively small amount of CHO in a portion, resulting in a small effect on blood glucose following consumption. For this reason, many prefer to use GL as a guide instead. GL considers the portion size as eaten and is calculated as GI x CHO in portion/100. For example, watermelon has a high GI of 70‐80, but as it is mainly water, a 100 g serving only contains 8 g CHO and so it has a low GL of 6. Published GI tables often contain a range of GIs for a given food (Foster‐Powell *et al*. 2002). This variation can be attributed to a range of factors including, for example preparation method, cooking time, storage conditions and variety/brand.

Furthermore, when CHO is consumed in the context of a mixed meal, the presence of fat and protein results in a lower glycaemic response and GI, than if the CHO was consumed on its own (Kim *et al*. 2019). Different food processing methods, such as cooling and reheating, also affect the glycaemic response to a meal. None of these factors, however, are currently taken into consideration when compiling GI tables for individual foods.

## Effects of cooling and reheating on starch fractions

When a starchy CHO is cooked and cooled, some of the gelatinised starch retrogrades to form RS3 (Chung *et al*. 2006). The amylose–amylopectin ratio determines how much retrograded starch remains on reheating, with retrograded amylose being more heat stable than retrograded amylopectin. Crystallinity in retrograded amylose is reduced by only 25% when heated to 90°C, whereas amylopectin crystallinity can be fully reversed by heating (Eliasson 2017) at temperatures as low as 55°C (Eerlingen & Delcour 1995). Cooling also produces changes in RDS and SDS with white rice displaying a reduction in RDS, along with an increase in SDS and RS after 10‐hour cold storage; reheating to 65°C reversed about 20% of the effects of cold storage, with increases in RDS and SDS observed (Lu & Monro 2016). Freshly cooked potato contains a high proportion of RDS; after chilling, this is significantly reduced and increases in both SDS and RS are observed (Mishra *et al*. 2008). In contrast to rice, reheating boiled potatoes reduces RS content almost to that observed in freshly cooked potatoes (Raatz *et al*. 2016). This may be explained by differences in amylose–amylopectin ratio, as rice has a higher amylose–amylopectin ratio (short grain, approx. 33:67; long grain, approx. 53:47) than potato which has a ratio ranging from 20:80 to 32:68 (Dipnaik & Kokare 2017; Karlsson *et al*. 2007; Nayak *et al*. 2014).

## Effects of cooling and reheating on glycaemic response

The effects of chilling and reheating a starchy food on glycaemic response vary between food types. Consuming precooked potatoes, cold, has been shown to elicit a lower glycaemic response than the equivalent freshly cooked potatoes (Leeman *et al*. 2005), whereas no differences were observed between freshly cooked and chilled–reheated boiled potatoes (Fernandes *et al*. 2005). In contrast, a reduction in glycaemic response was observed following a reheated pasta meal in comparison with freshly cooked, with no differences observed for a chilled pasta meal (Hodges *et al*. 2020). Similarly, chilled and reheated white rice elicited a lower glycaemic response than freshly cooked white rice (Sonia *et al*. 2015). These differences may be attributed to a range of factors, including differences in amylose–amylopectin ratio, cooking and storage methods and amount of starch gelatinisation and retrogradation.

## Hepatic glucose production

Another contributor to the extent of postprandial glycaemia is the degree to which hepatic glucose production is suppressed in response to the ingestion of glucose‐containing carbohydrate. This can only be measured using stable isotope techniques, which can measure the flux of glucose from the liver (endogenous glucose production) into the peripheral circulation as well as glucose arriving from the test meal (Fielding *et al*. 2020). In order to truly represent the metabolic fate of glucose molecules chemically linked in starch, the glucose must be intrinsically labelled with stable isotope atoms.

## Impact of lipids on starch digestibility and glycaemic response

Lipids may act to reduce starch digestibility in several ways. They may act as a physical barrier, surrounding the starch granules, inhibiting enzymatic digestion and reducing granule swelling (Debet & Gidley 2006). They may also complex with amylose, forming RS5 (Holm *et al*. 1983). To date, much of the evidence for RS5 formation comes from laboratory studies, using free fatty acids. There has been little research into RS5 formation using triacylglycerol, the form most lipid takes in food. It is thought that the structure of triacylglycerol restricts complex formation; however, evidence of weak complex formation has been reported for corn oil (Ai *et al*. 2013), and evidence of complexing index (CI), a marker of RS5 formation, has also been reported in breads made with various common fats and oils (Lau *et al*. 2016). Fatty acid composition affects complex formation, with chain length and degree of saturation affecting the amount of complex formation and complex melting point (Kaur & Singh 2000; Soong *et al*. 2013). Lau *et al*. reported different CIs for breads made with different fats; they reported higher CI for breads made with coconut oil and olive oil than those made with butter and grapeseed oil (Lau *et al*. 2016). Although CI was correlated with glycaemic response in their postprandial study, there were no significant differences in glycaemic response between breads. Reed *et al*. measured starch hydrolysis rates and total RS content of several varieties of rice, cooked in different ways (Reed *et al*. 2013). They reported less RDS and SDS, and more RS in stir‐cooked rice (no oil) after cold storage, than in both freshly cooked steamed rice (no oil) and freshly cooked stir‐fried rice (with oil). They also reported less RDS and more RS in stir‐fried rice (with oil) after cold storage, than in all other methods, demonstrating a cumulative effect of cold storage and oil.

These results suggest a potential for beneficial effects on the glycaemic response from these cooking methods; however, to date there has been little research in this area. Our own pilot data (Robertson *et al*. 2020) found a reduction in the glycaemic response to a pasta meal (pasta coated in oil with tomato sauce), consumed cold, in comparison with the identical freshly cooked meal and further attenuation of the glycaemic response when the chilled pasta meal was reheated and consumed hot.

## Resistant Starch Production and Glucose Release from Pre‐Prepared Chilled Food: The SPUD Project

This will be a joint project between the Universities of Surrey and Cambridge, involving Branston Ltd as a project partner and Iso*Life* (Netherlands) for expert intrinsic stable isotope labelling of a food – starch. The project will aim to progress our pilot work, utilising a different CHO food. We have chosen the potato as a CHO, which is widely consumed in the UK. In 2017/2018, annual UK consumption of potatoes (fresh and processed) was estimated at 669 g per person per week, compared to 527 g for bread, and 582 g for cereal and cereal products, a category which includes rice and pasta (Defra 2020). We will test mashed potato as it is commonly prepared with butter or oil, which will allow us to incorporate lipid into the meal in a natural fashion. Furthermore, the mashing process will minimise the effects of chewing differences/particle size on starch digestibility (Cañas *et al*. 2020).

**Figure 1:**
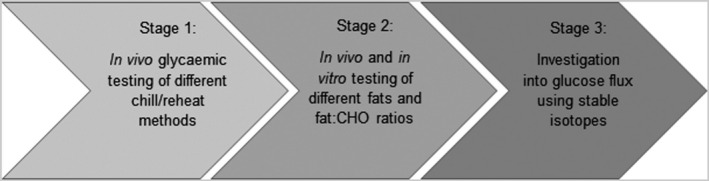
Schematic overview of the SPUD project.

The project will involve a mix of human and *in vitro* studies, with the results of each stage informing the next. Stage 1 will test the effects of different storage temperatures and reheating methods on the glycaemic response to determine the optimum combination. This optimum method will be taken forward into Stage 2, which will test the effects of different fats on the glycaemic response. Running parallel to this will be an *in vitro* study, measuring RDS, SDS and total RS formation in each of the test meals, using two different fat–CHO ratios. The results of this will inform Stage 3, which will compare freshly cooked mashed potato incorporating the optimum fat–CHO ratio from Stage 2 with an identical reheated meal. This study will utilise a dual stable‐isotope methodology, allowing detailed glucose flux modelling. The stages are summarised in Figure 1.

### Stage 1

**Figure 2:**
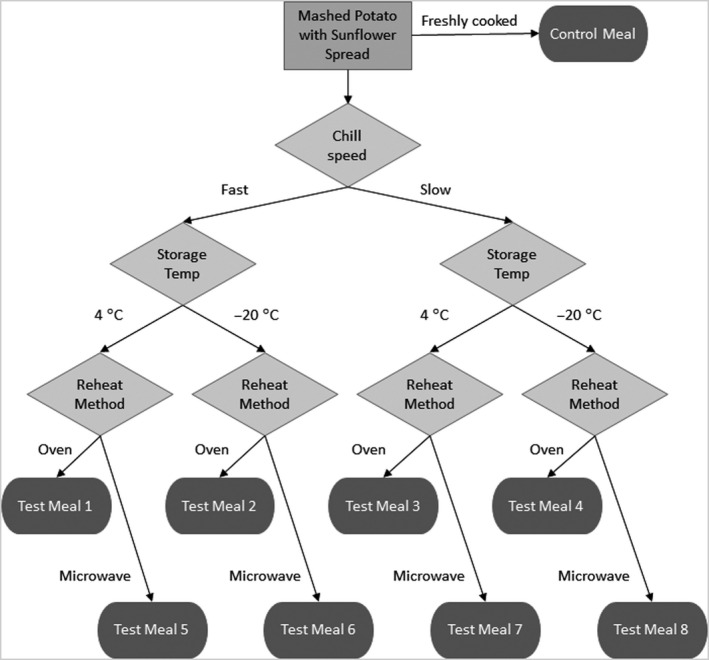
Schematic overview of the test meal preparation protocol for Stage 1 of the SPUD project.

The objective of Stage 1 is to carry out a human study, analysing the blood glucose response to consumption of mashed potato under different cook–chill–reheat conditions, with the aim of determining the optimum chill/reheat method to minimise the postprandial glucose excursion. Factors tested will be chill speed (room temperature vs. quick chill), storage temperature (4°C vs. −20°C) and reheating method (microwave vs. conventional oven). An overview of the study design is presented in Figure 2.

Participants will each attend five study days, consuming a different meal at each visit. Capillary blood samples will be taken for 2 hours postprandially. The samples will be immediately separated, and the plasma stored at −20°C for batch analysis of glucose and insulin at the end of the study. Participant inclusion criteria are healthy males and females, aged 18‐55 years, normal weight (BMI 18.5‐25 kg/m^2^) and not taking any prescription medication, apart from contraceptives, within the last 3 months; exclusion criteria include pregnant or lactating females, people following a weight reducing diet and people with previous or current medical conditions such as heart disease, diabetes, liver disease, endocrine diseases and gastrointestinal diseases. The study will utilise an incomplete block design, with each participant consuming the control meal (freshly cooked mashed potato) and four randomly allocated test meals. In order to test each meal combination 8 times, *n* = 16 subjects are required, at a power of 90% to detect a difference in glucose IAUC of 13% over 2 hours or a difference of 11% at 80% power. The test meal producing the greatest attenuation of the glucose response in comparison with the control meal, will be carried forward to Stage 2.

### Stage 2

In Stage 2, the effects of three commercially available fat spreads, of differing fatty acid composition, will be tested. In a human study, 10 participants will be recruited to attend six study days. The participant inclusion/exclusion criteria are as per Stage 1. At each visit, they will consume one of the test meals, in a randomised crossover order, and provide capillary blood samples for 2 hours postprandially. Samples will be stored and analysed as per Stage 1. They will each consume all the test meals over the course of the study. Comparisons will be made between fat spreads, for the postprandial glucose, and insulin responses, for both the freshly cooked and reheated cooking methods. The differences in glucose and insulin response between freshly cooked and reheated methods will also be directly compared for each individual spread. An *in vitro* study will measure the RDS, SDS and RS content of meals prepared according to the protocol for the human study but incorporating the fat spread at two different concentrations: 5 g fat/100 g potato and 10 g fat/100 g potato. The test meals for both studies are summarised in Table 2. The spread type that produces the greatest difference in glycaemic response between freshly cooked and reheated meals will be carried forward to Stage 3.

**Table 2:** Test meal composition for Stage 2 of the SPUD project

Test meal	Cooking method	Spread type	amount of fat per 100 g potato (g)	Study
1	F	Butter	10	B
2	F	Olive oil spread	10	B
3	F	Sunflower spread	10	B
4	R	Butter	10	B
5	R	Olive oil spread	10	B
6	R	Sunflower spread	10	B
7	F	Butter	5	I
8	F	Olive oil spread	5	I
9	F	Sunflower spread	5	I
10	R	Butter	5	I
11	R	Olive oil spread	5	I
12	R	Sunflower spread	5	I

F, freshly cooked mashed potato; R, reheated mashed potato; B, both human and in vitro; I, in vitro only.

### Stage 3

The aim of this stage is to perform a postprandial study, using the optimum cook–chill–reheat method and fat spread from previous stages compared to freshly prepared mashed potato. It will utilise a dual stable isotope methodology to quantitate glucose flux parameters (glucose uptake from the gut, hepatic glucose uptake and peripheral glucose uptake).

Twelve participants will be recruited, each attending two study days. The participant inclusion/exclusion criteria are as per Stage 1. On one day, they will consume the freshly cooked mashed potato meal, and on the other, they will consume the identical reheated meal; the meal order will be randomised. Each meal will consist of intrinsically labelled [U‐^13^C] potato, produced to > 97 % enrichment, by Iso*Life* (https://isolife.nl/), diluted to 5% with non‐labelled potato, along with the fat spread selected from Stage 2. The use of intrinsic labelling will allow examination of the effects of changes to the food matrix on the glycaemic response.

On each study day, the participant will receive a labelled [6,6‐^2^H_2_]glucose infusion, via a cannula inserted into an antecubital vein. This will continue at a steady rate for 2 hours, to enable equilibrium to be reached. They will then consume the labelled mashed potato. The [6,6‐^2^H_2_]glucose infusion will continue for the next 6 hours, but the rate will be varied to mimic endogenous glucose production. Venous blood samples will be taken via a second cannula inserted into an antecubital vein in the participant’s other arm. In this method, the [6,6‐^2^H_2_]glucose tracer is used to measure endogenous glucose production, whilst the [U‐^13^C]glucose consumed in the meal is used to quantitate the rate of appearance of oral glucose.

Plasma glucose samples will be derivatised for gas chromatography mass spectrometry, and ions *m/z* 319.2 (M + 0), 321.2 (M + 2) and 323.2 (M + 4) measured for determination of tracer–tracee ratios of [6,6‐^2^H_2_]glucose and [U‐^13^C]glucose. Mathematical modelling based on the Bayes method will be used to estimate endogenous glucose production, rate of glucose appearance from meal and rate of glucose disposal. This methodology has been validated previously (Haidar *et al*. 2012a, 2012b). Gut hormones (insulin, glucose‐dependent insulinotropic polypeptide and glucagon‐like peptide 1) and blood lipids (non‐esterified fatty acids and triacylglycerol) will also be measured as additional markers demonstrating the delay and/or displacement of gastrointestinal nutrient absorption.

## Conclusions

There are new evidence‐based guidelines concerning the recommended intakes of free sugars (<5% energy) and total CHO (~50% energy) in the UK diet; however, there remains no direct advice regarding dietary starch, despite this potentially contributing up to 45% of daily energy requirements. Starchy foods are unique within the diet in that they are always processed in some way before consumption. However, both manufacturers and consumers know very little about the impact of this processing on our health, for what represents a very large part of the UK diet. With the *SPUD* project, we aim to examine the effects on glycaemia of domestic food processing techniques using the potato, as model CHO, and to further investigate the potential underlying mechanisms of action. Determining the relationship between food processing, starch structure and glucose metabolism may be key in understanding their role in human health. As cooking, chilling and reheating carbohydrates is an everyday occurrence for many, such information would be important not just for the consumer, but for the food industry in terms of producing, marketing and labelling healthier alternatives for consumers.

## Conflict of interest

The authors declare no conflict of interest.
